# Tcf7 Is an Important Regulator of the Switch of Self-Renewal and Differentiation in a Multipotential Hematopoietic Cell Line

**DOI:** 10.1371/journal.pgen.1002565

**Published:** 2012-03-08

**Authors:** Jia Qian Wu, Montrell Seay, Vincent P. Schulz, Manoj Hariharan, David Tuck, Jin Lian, Jiang Du, Minyi Shi, Zhijia Ye, Mark Gerstein, Michael P. Snyder, Sherman Weissman

**Affiliations:** 1 Department of Genetics, Stanford University School of Medicine, Stanford, California, United States of America; 2 Genetics Department, Yale University School of Medicine, New Haven, Connecticut, United States of America; 3 Department of Pediatrics, Yale University School of Medicine, New Haven, Connecticut, United States of America; 4 Department of Pathology, Yale University School of Medicine, New Haven, Connecticut, United States of America; 5 Computer Science Department, Yale University, New Haven, Connecticut, United States of America; 6 College of Preventive Medicine, Third Military Medical University, Chongqing, China; 7 Program in Computational Biology and Bioinformatics, Yale University, New Haven, Connecticut, United States of America; 8 Molecular Biophysics and Biochemistry Department, Yale University, New Haven, Connecticut, United States of America; The University of North Carolina at Chapel Hill, United States of America

## Abstract

A critical problem in biology is understanding how cells choose between self-renewal and differentiation. To generate a comprehensive view of the mechanisms controlling early hematopoietic precursor self-renewal and differentiation, we used systems-based approaches and murine EML multipotential hematopoietic precursor cells as a primary model. EML cells give rise to a mixture of self-renewing Lin-SCA+CD34+ cells and partially differentiated non-renewing Lin-SCA-CD34− cells in a cell autonomous fashion. We identified and validated the HMG box protein TCF7 as a regulator in this self-renewal/differentiation switch that operates in the absence of autocrine Wnt signaling. We found that *Tcf7* is the most down-regulated transcription factor when CD34+ cells switch into CD34− cells, using RNA–Seq. We subsequently identified the target genes bound by TCF7, using ChIP–Seq. We show that TCF7 and RUNX1 (AML1) bind to each other's promoter regions and that TCF7 is necessary for the production of the short isoforms, but not the long isoforms of RUNX1, suggesting that TCF7 and the short isoforms of RUNX1 function coordinately in regulation. *Tcf7* knock-down experiments and Gene Set Enrichment Analyses suggest that TCF7 plays a dual role in promoting the expression of genes characteristic of self-renewing CD34+ cells while repressing genes activated in partially differentiated CD34− state. Finally a network of up-regulated transcription factors of CD34+ cells was constructed. Factors that control hematopoietic stem cell (HSC) establishment and development, cell growth, and multipotency were identified. These studies in EML cells demonstrate fundamental cell-intrinsic properties of the switch between self-renewal and differentiation, and yield valuable insights for manipulating HSCs and other differentiating systems.

## Author Summary

The hematopoietic system has provided a leading model for stem cell studies, and there is great interest in elucidating the mechanisms that control the decision of HSC self-renewal and differentiation. This switch is important for understanding hematopoietic diseases and manipulating HSCs for therapeutic purposes. However, because HSCs are currently unable to proliferate extensively in vitro, this severely limits the types of biochemical analyses that can be performed; and, consequently, the mechanisms that control the decision between early-stage HSC self-renewal and differentiation remain unclear. Murine bone marrow derived EML multipotential hematopoietic precursor cells are ideal for studying the switch. EML cells can grow in large culture and give rise to a mixture of self-renewing Lin-SCA+CD34+ cells and partially differentiated non-renewing Lin-SCA-CD34− cells in a cell autonomous fashion. Using RNA–Sequencing and ChIP–Sequencing, we identified and validated the HMG box protein TCF7 as a regulator in this switch and find that it operates in the absence of canonical Wnt signaling. Together with RUNX1, TCF7 regulates a network of transcription factors that characterize the CD34+ cell state. This work serves as a model for studying mechanisms of autonomous and balanced cell fate choice and is ultimately valuable for manipulating HSCs.

## Introduction

Stem cells are characterized by the ability to both self renew and undergo cell differentiation. Understanding the mechanisms that control the switch between renewal and differentiation is a fundamental and important problem in stem cell biology. It is likely that many key components including signaling molecules and transcription factors are involved in this process. Although a few key components that influence the switch have been found [1], [2], [3], [4], it likely that many others exist. Identification of such components and elucidation of how they function is critical for understanding this developmental switch.

Blood-forming hematopoietic stem cells (HSCs) are one of the best-characterized stem cells, and are valuable for studying self renewal and differentiation [5], [6]. HSCs exist in adult bone marrow, and can self-renew and differentiate into more than ten distinct mature blood cell lineages after transplantation *in vivo* [7]. Understanding the mechanisms that regulate differentiation of HSCs into the different cell types is expect to be important for understanding hematopoietic diseases and manipulating HSCs for therapeutic purpose. However, because HSCs are currently unable to proliferate extensively *in* vitro without losing their “stemness”, large cultures cannot be produced [8]. This severely limits the types of biochemical and genomic analyses that can be performed, and consequently, the mechanisms that control the decision between early-stage HSC self-renewal and differentiation remain unclear.

The mouse (*Mus musculus*) EML (Erythroid, Myeloid, and Lymphocytic) multipotential hematopoietic precursor cell is an ideal system for studying the molecular control of early hematopoietic differentiation events. EML cells are derived from mouse bone marrow cells that have been transfected with a retrovirus expressing a dominant negative retinoic acid receptor and were subsequently cultured in the presence of stem cell factor (SCF). These cells can be re-derived or repeatedly cloned and still retain their multipotentiality [9], [10], [11]. The ability of EML cells to propagate extensively in medium containing SCF makes them ideal for biochemical and genetic assays as well as high throughput functional screens [7], [12]. Phenotypically, EML cells express many of the cell surface markers characteristic of hematopoietic progenitor cells, including SCA1, CD34, and c-KIT. Functionally, when treated with different growth factors, such as SCF, IL-3, GM-CSF, and EPO, EML cells can differentiate into distinct cell lineages including B-lymphocyte, erythrocyte, neutrophil, macrophage, mast cell, and megakaryocyte lineages [9]. Unlike maturation of human promyelocytic cell lines, such as NB4 and HL60, EML cell derivatives develop into mature neutrophils with segmented nuclei and azurophilic granules [9]. Thus, EML cells are biologically relevant for normal hematopoiesis.

Interestingly, in culture the Lin-SCA+CD34+ subpopulation of EML cells, which can be isolated by magnetic-activated cell sorting (MACS) beads or Fluorescence Activated Cell Sorting (FACS), gives rise in culture to a mixed population containing similar numbers of self renewing Lin-SCA+CD34+ precursor cells and partially differentiated Lin-SCA-CD34− cells (henceforth referred to as CD34+ and CD34− cells, respectively) [13]. Although the two populations resemble each other morphologically, only the CD34+ population propagates in SCF-containing media, while the CD34− cells do not self-renew in SCF; instead, their growth requires the cytokine IL-3 [13]. The closest normal analogs of CD34+ cells are short-term (ST)-HSC or multipotent progenitors (MPP). Similar to short-term (ST)-HSC, CD34+ cells are capable of self-renewal; like MPP, when treated with cytokines such as IL-3, CD34+ cells can give rise to CD34− cells with more restricted potential. A number of erythroid genes, such as α- and β-hemoglobin, Gata1, Epor (erythropoietin receptor), and Eraf (erythroid associated factor), as well as mast cell proteases are expressed at a significantly higher level in the CD34− cell population than CD34+ cells [13], [14]. This indicates that the CD34− cells were, at minimum, differentiated into a state with prominent erythroid potential.

The ability of CD34+ cells to both differentiate and self-renew in suspension culture in the absence of any anatomical niche or other cell type suggests that CD34+ cells are regulated by a tightly controlled endogenous mechanism that guides the generation of the variety and relative abundance of the cell types in culture. Understanding the molecular events that regulate the transition between the two types of putative precursors in the EML multipotent hematopoietic cell line will give insights to the fundamental mechanisms of autonomous balanced selection of alternative cell fates available for stem cells and intermediate-stage cancer precursor cells [7].

What is the mechanism that regulates the decision between the two types of precursor cells? One possible mechanism is by modulating the levels of key transcriptional regulators. This hypothesis is suggested by the findings that Pu.1 or Gata1 play a determining role in downstream hematopoietic lineage decisions [15], [16]. Higher Pu.1 expression switches the differentiation to the myeloid lineages [16], [17] whereas Gata1 shifts cells towards the erythroid lineage. In light of this, we examined transcription factors that were significantly up-regulated in CD34+ cells relative to CD34− cells using RNA sequencing (RNA-Seq) and found *Tcf7* (also referred to by the symbol *Tcf1*) to be the most strongly up-regulated transcription factor. TCF7 is a member of a family of HMG box containing factors that are known to associate with beta-catenin in the nucleus to mediate Wnt signaling [18], [19], [20]. Wnt signaling has been implicated in hematopoietic stem cell and precursor maintenance and affects the decision between self-renewal and differentiation [21], [22], [23], [24] although its role in EML cells is not yet defined. It has been reported that TCF7 plays a role in B cell and T cell development and is a possible co-regulator in mouse embryonic stem cells, but TCF7 has not been noted for its function in earlier decisions in hematopoietic development [25], [26]. The binding motifs of the TCF family of transcription factors are significantly enriched among genes that are expressed at a higher level in CD34+ than in CD34− cells. Therefore, we hypothesized that TCF7 is one of the key transcription factors that control a transcriptional regulatory network determining the choice between EML cell self-renewal and differentiation.

We identified *in vivo* binding targets of TCF7 using ChIP-Seq (chromatin immunoprecipitation in combination with high-throughput sequencing). We found TCF7 binds to its own promoter and the promoter of *Runx1 (Aml1)*, a developmental determinant in hematopoietic cells that is best known for its critical role in hematological malignancies [27], [28]. RUNX1 and TCF7 were found to bind to each other's promoters and a large number of common target genes are bound by RUNX1 and TCF7. Analysis correlating gene expression and transcription factor binding data suggests that TCF7 is necessary to maintain cells in the undifferentiated state. We validated this hypothesis by knock-down of *Tcf7* expression. Finally, through network analysis, we found that TCF7 and RUNX1 bind and regulate a network of up-regulated transcription factors in the CD34+ cells which characterize the self-renewal property of the CD34+ cells. Importantly, in EML cells TCF7 functions in the absence of autocrine Wnt signaling. Our results thus elucidate novel components and mechanisms that control stem cells renewal and differentiation.

## Results

### Differentially expressed genes in CD34+ and CD34− EML precursor cells identified using RNA–Seq

**Figure 1:**
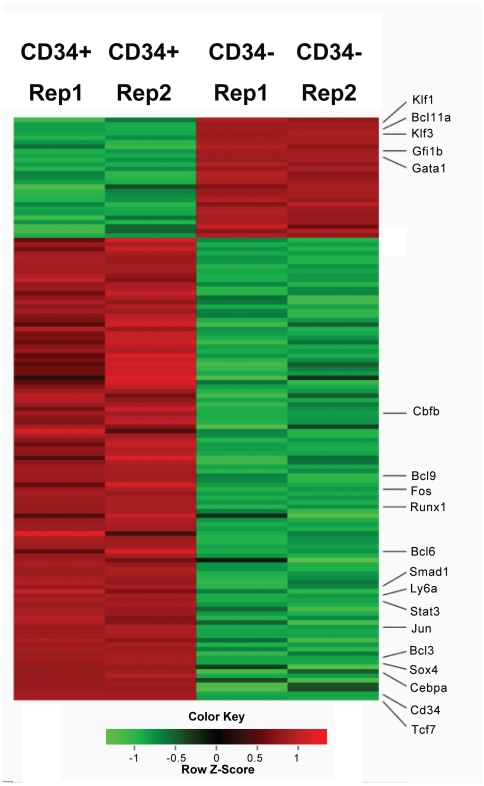
Heatmap display of transcription factors that differential expressed (>1.5 fold) between Lin-CD34+ cell and Lin-CD34− cells. Two replicas were shown for each cell type. Red color represents up-regulated genes and green color represents down-regulated genes. Genes mentioned in the text are labeled. *Cd34* and *Ly6a (Sca1)* are cell surface markers. See “Methods” for the calculation of Z scores.

Global identification of gene expression can provide significant insight into molecules important for the self-renewal and differentiation decisions in EML cells. Differential gene expression between CD34+ and CD34− cells was first studied using cDNA microarrays [13]. As cDNA microarrays do not cover the entire transcriptome, we decide to investigate the gene expression profiles of CD34+ and CD34− cells using the RNA-Seq technology. We generated 35 nt single end and long 75 nt paired-end reads (sequence reads from both ends of cDNA fragments) using Illumina technology. Although the overall patterns of mRNA levels are similar in CD34+ and CD34− cells, the expression levels of a limited number of transcription factors differ in the two cell populations (Figure 1; Dataset S1).) Notably, the expression level of *Tcf7* was found to be over 100 fold higher in the CD34+ cells relative to CD34− cells, where it is very low. In fact *Tcf7* is as strongly regulated as the cell surface marker *Cd34* itself.

*Tcf7* is a member of the T-cell factor family including *Tcf3*, an essential component of the core regulatory network controlling the balance between pluripotency and differentiation in mouse embryonic stem (mES) cells [29]. However, the *Tcf3* expression level is very low in EML cells, and neither *Tcf3* nor other TCF family members displayed the remarkable differential gene expression observed for *Tcf7*. Other interesting transcription factors that we found to be up-regulated in CD34+ cells include: *Sox4*, *Jun*, *Stat3*, *Smad1*, *Cebpa*, *Bcl6*, *Bcl3*, *Bcl9*, *Fos*, *Runx1* and *Cbfb* (Figure 1). Consistent with the microarray study, we also found that transcription factors involved in erythroid differentiation, such as *Gata1*, *Gfi1b Klf1* and *Klf3*, are up-regulated in CD34− cells (Figure 1).

### Enriched functional categories and motif analysis of differentially expressed genes

We next examined the functional categories of differentially expressed transcription factors in either CD34+ or CD34− cells using Ingenuity Pathway Analysis software (IPA). The top three significant categories in Molecular and Cellular Functions are “Gene Expression”, “Cellular Development”, and “Cellular Growth and Proliferation”, whereas the top three significant categories in Physiological System Development and Function are: “Hematological System Development and Function”, “Hematopoiesis”, and “Tissue Morphology” (Table S1). These categories are consistent with the suggestion that the switch from CD34+ to CD34− cells represents a developmental process towards a less proliferative and more differentiated state.

To further identify the potential transcription factors that control the group of genes up-regulated in CD34+ cells, we performed Distant Regulatory Elements analysis (DiRE http://dire.dcode.org/). DIRE analyzes not only the proximal promoter regions but also the full gene locus including intergenic, promoter, intronic and UTR (upstream untranscribed regions). Among the DNA sequence motifs that were enriched in up-regulated genes (>1.5 fold) in CD34+ cell in comparison to CD34− cells, were binding motifs for members of the TCF family of transcription factors (Figure S1).

### Analysis of gene expression of Wnt pathway components showed minimum endogenous Wnt signaling in EML cells

TCF7 has previously been studied as a partner with nuclear beta-catenin, which serves as a downstream transcriptional activator in response to external Wnt signaling. TCF7 potentially acts as a transcriptional repressor in the absence of beta-catenin [18], [19]. We therefore examined the data for expression of components of the Wnt signaling pathway. Internal components of the pathway such as *Apc*, *Axin1*, *Bcl9*, *Daam1*, *Gsk3a* and *Gsk3b* were expressed at the mRNA level (Table S2) in both CD34+ and CD34− cells. Activation of the canonical Wnt pathway is achieved primarily through various Wnt ligands binding to LRP5/6 and/or frizzled (FZD) receptors. Of the components of canonical Wnt receptors, a moderate level of *Lrp5/6*, *Fzd2* and *Fzd7* mRNA were detected by either RNA-Seq or Illumina microarrays analysis, and even lower levels of mRNA for other Frizzled genes. Based on RNA-Seq data, the Wnt ligands were absent with the exception of *Wnt9a* and *Wnt10a* which were present in trace amounts (Table S2). The Illumina microarray also reported the presence of low levels of *Wnt10a* mRNA. To further test for the presence of *Wnt10a* mRNA, four sets of PCR primers were designed that crossed introns, and could distinguish between genomic DNA and spliced cDNA. Each set of primers gave the anticipated band from mouse embryonic fibroblast cDNA, but none showed any band of the specific product using CD34+ cell cDNA (data not shown).

Despite the fact *Tcf7* expression is abundant; the absence or very low level of mRNA for Wnt ligands suggests that EML cells have little or no endogenous Wnt signaling. To test this possibility we utilized a *Tcf/Lef* GFP reporter to monitor Wnt activity in EML cells. The *Tcf/Lef* reporter is under the control of a minimum CMV promoter fused in tandem to *Tcf/Lef* transcriptional response elements. EML cells containing the positive control CMV-GFP construct showed uniform robust expression in EML cells (Figure S2). By contrast, cells containing the *Tcf/Lef* reporter failed to express GFP. To circumvent the possibility that the *Tcf/Lef* reporter is non functional in EML cells, we incubated the cells with either LiCl (50 mM), Wnt3a or Wnt5a (400 uM) for 24 hours. LiCl, a GSK3beta-inhibitor, has been previously used to activate the Wnt pathway [30], [31]. Upon stimulation with LiCl we noted a significant increase in GFP expression (52 fold enrichment when MOI = 5; 85 fold enrichment when MOI = 10) suggesting that cells are capable of receiving and activating the *Tcf/Lef* reporter (Figure S2). *Tcf/Lef* reporter activation using Wnt ligands was also observed, although it was lower than LiCl activation (data not shown) consistent with the findings that EML cells have low levels of frizzled receptors. These data demonstrate that while the cells are capable of response to activation of the internal Wnt response system, there is no endogenous Wnt signaling in EML cells detectable with the commonly used Wnt reporter system.

### Identification of TCF7 in vivo binding targets

**Figure 2:**
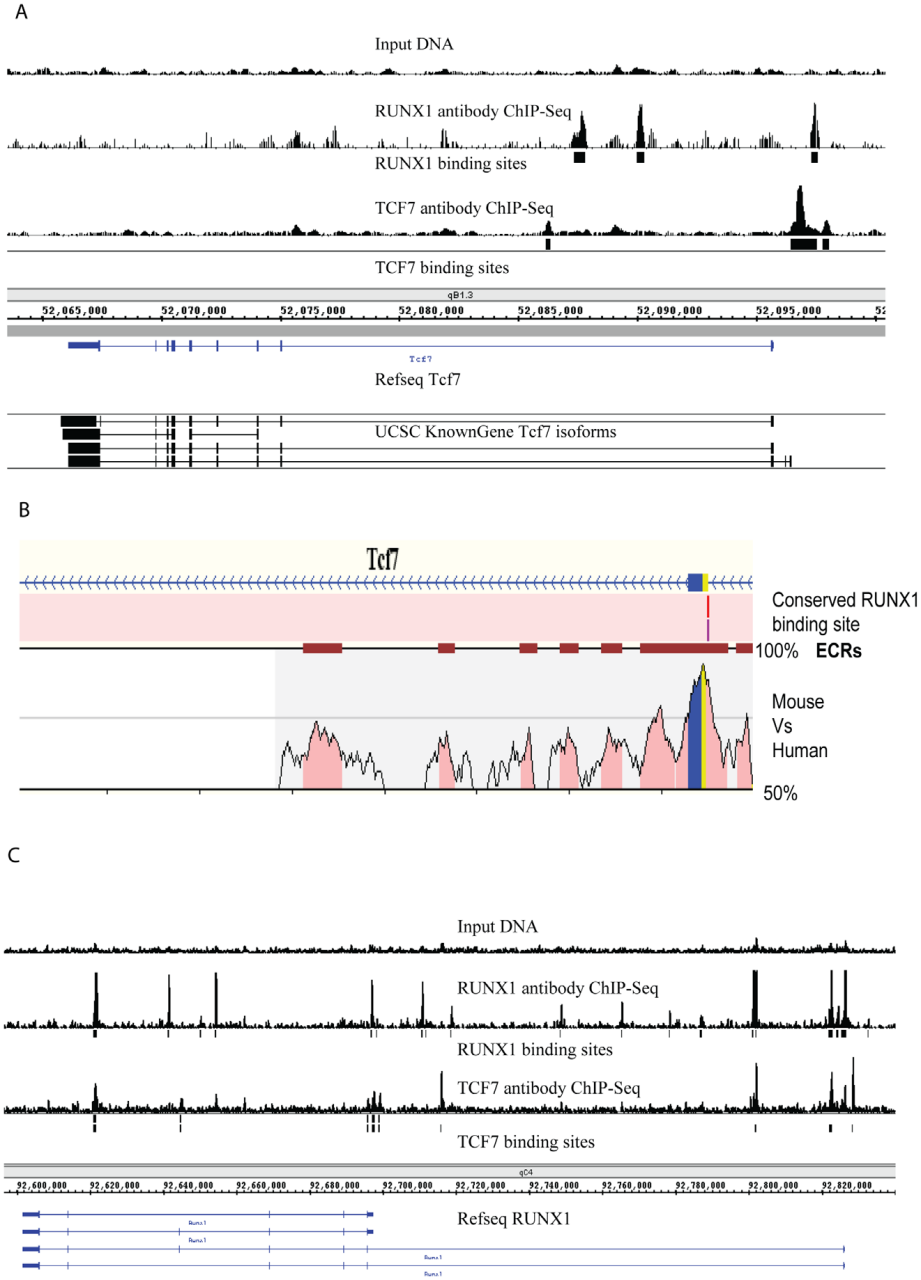
Identification of transcription factor binding targets using ChIP–Sequencing. (A) *Tcf7* is bound by both itself and by RUNX1 (AML1). Peaks indicate ChIP-Sequencing signal. Input genomic DNA serves as the negative control. The “binding sites” tracks (black vertical bars) show the transcription factor binding loci determined using the PeakSeq program (normalized against genomic input DNA; q-value<0.001). Data are visualized in Integrated Genome Browser (B) Identification of evolutionarily conserved RUNX1 binding sites at *Tcf7* promoter region using REGULATORY VISTA. The graph shows conserved and aligned AML1/RUNX1 transcription factor binding sites between mouse and human genomes using a matrix similarity score of 1 (the most stringent). Two versions of the AML1 binding sites were found (AML1 and AML_Q6). The *ECRs: Evolutionary Conserved Regions are indicated by deep red blocks. The degree of conservation (50%–100%) is indicated by the height of the peaks. Coding region is sown by blue and UTR is shown by yellow. (C) *Runx1* promoter is bound by both TCF7 and itself.

In order to better understand how TCF7 may be involved in the switch from self-renewing CD34+ cells to partially differentiated CD34− cells, we identified the *in vivo* binding sites for TCF7 using ChIP-Seq [32], [33]. We also performed PolII ChIP-Seq to follow genes that were activated or poised for activation. ChIP-Seq experiments identified 9696 TCF7 binding sites with a q-value (Benjamini Hochberg corrected p-value)< = 0.001 (Dataset S2). The binding sites were mapped to RefSeq genes in the UCSC mm9 database (genome.ucsc.edu). The binding sites were assigned to a particular gene if the peak was present within 3 kb upstream of the transcription start site or inside of the gene (including both exonic and intronic regions)(Dataset S4). ∼85% of TCF7 binding peaks were assigned to known genes using this approach. These sites were mapped to 7976 TCF7 target genes. Among the interesting binding sites was the presence of TCF7 at its own promoter region in CD34+ cells (Figure 2A), raising the possibility that TCF7 regulates its own transcription through an autoregulatory feedback loop. qPCR experiments verified the enrichment of TCF7 binding at the *Tcf7* promoter region in CD34+ cells using TCF7 antibody compared to IgG immunoprecipitation.

To explore the biological processes that are regulated by TCF7, the functional categories associated with TCF7 target genes were examined based on annotations in the Gene Ontology (GO) database [34]. Enriched GO categories were identified and displayed using the BiNGO program (http://www.psb.ugent.be/cbd/papers/BiNGO/) (Figure S3) [35]. Genes associated with regulation of transcription were highly enriched in TCF7 targets, consistent with our hypothesis that TCF7 functions as a key transcription regulator in the decision of EML cell self-renewal and differentiation. Other significantly enriched functional categories include cell development and differentiation, metabolic processes, and signaling.

### RUNX1 functions coordinately with TCF7 in regulating target genes

In an effort to understand how the expression of *Tcf7* itself is controlled to regulate hematopoietic development, we examined the promoter of *Tcf7* for other potential regulators. Evolutionarily conserved RUNX1 (AML1) binding sites were identified at the *Tcf7* promoter region (Figure 2B) using the software REGULATORY VISTA (http://ecrbrowser.dcode.org). In addition, the *Runx1* expression pattern is consistent with *Tcf7* regulation, i.e. although expressed in both CD34+ and CD34− cells, *Runx1* mRNA is up-regulated 3.7 fold in the CD34+ cells as shown by RNA-Seq data (Figure 1). Using a previously validated anti-RUNX1 rabbit polyclonal antibody [36], ChIP-Seq experiments identified 21932 RUNX1 binding sites with a q-value (Benjamini Hochberg corrected p-value)< = 0.001 (Dataset S3). These sites were mapped to 5393 RUNX1 target genes (Dataset S4). We performed de novo binding motif search among sequences bound in TCF7 and RUNX1 ChIP-Seq experiments, and found they overlap well with the known motifs (from Jaspar and Transfac databases) of RUNX1 and TCF7 (Figure S4). We found RUNX1 binds to the *Tcf7* promoter (Figure 2A), as well as its own promoter. Therefore, it is likely that autoregulation of RUNX1 contributes to the regulation of *Tcf7*. Furthermore, TCF7 also binds to the *Runx1* gene (Figure 2C). Since both transcription factors bind to their own respective promoters as well as to each other, one intriguing possibility is that TCF7 and RUNX1 may co-regulate each other in a feed-back loop.

**Figure 3:**
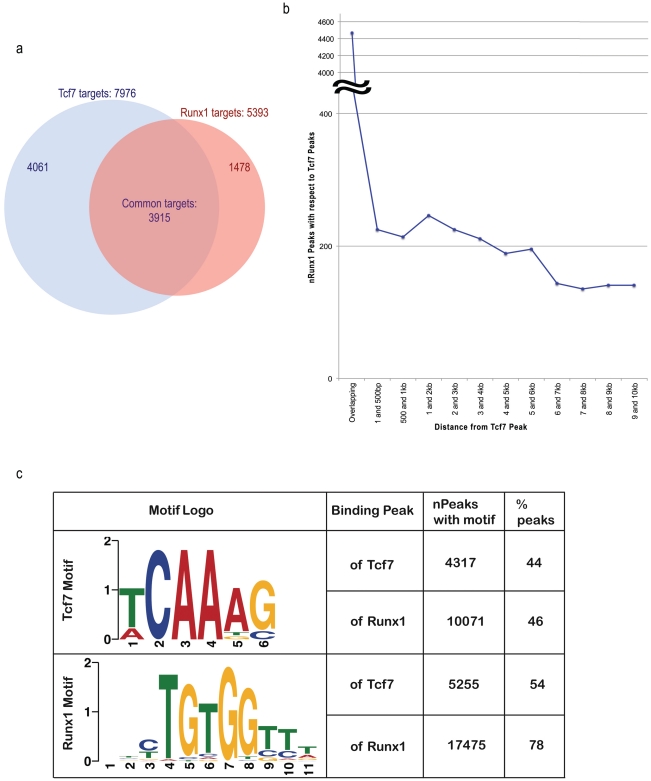
Binding site analyses suggest RUNX1 functions coordinately with TCF7 in regulating target genes. (A) In the Venn diagram, the blue circle represents TCF7 target genes and red circle represents RUNX1 target genes. 3915 target genes (72% of all RUNX1 targets and 49% of all TCF7 target genes) are common to each other. The hypergeometric p-value of the intersection (compared to the entire UCSC known gene collection 24901 genes) is 6.72294e-56. (B) The graph represents the distribution of the distance of the RUNX1 peak to the TCF7 peaks. (C) The distribution of TCF7 binding motifs and RUNX1 motifs in binding regions of both factors are presented. Since TCF7 had two versions of the motifs, non-duplicate union of occurrence of both motifs was indicated in the table. The sequence of one (version) of the motif logos is shown in the table.

We used three approaches to test whether RUNX1 functions coordinately with TCF7 in regulating target genes. In the first approach we compared the TCF7 ChIP-Seq and RUNX1 ChIP-Seq data to identify the overlapping set of target genes for the two transcription factors. Among 7976 TCF7 target genes (Figure 3A, blue circle in the Venn diagram) and 5393 RUNX1 target genes (Figure 3A, red circle), 3915 target genes are in common (72% of all RUNX1 targets and 49% of all TCF7 target genes are common; see Figure 3A). The hypergeometric p-value of the intersection is 6.72294e-56. Thereby, TCF7 and RUNX1 binding target lists showed a statistically significant overlap. In the second approach, the proximity of RUNX1 and TCF7 binding peaks were analyzed. The distance of the nearest RUNX1 peak to each of the TCF7 peak was identified. RUNX1 peaks either overlap with, or are within 500 nt upstream or downstream of 4691 of the 9696 TCF7 peaks (48%; Figure 3B). The third approach used motifs to which the factors are known to bind. Two versions of the known motifs for TCF7 and two known motifs for RUNX1 were obtained from Jaspar and Transfac databases. We assessed the occurrence of each of these motifs within the experimentally identified binding sites of TCF7 and RUNX1. Within TCF7 peaks, at least 44% of the peaks contained one version of the TCF7 motif and 54% of the peaks contained at least one RUNX1 motif. Within the RUNX1 peaks, 46% had a TCF7 motif and 78% had a RUNX1 motif (Figure 3C). Based on all above-mentioned analyses, we conclude TCF7 and RUNX1 bind to a large number of shared target genes and likely function coordinately in regulation of gene expression.

### TCF7 primarily binds to the genes up-regulated in CD34+ cells

**Figure 4:**
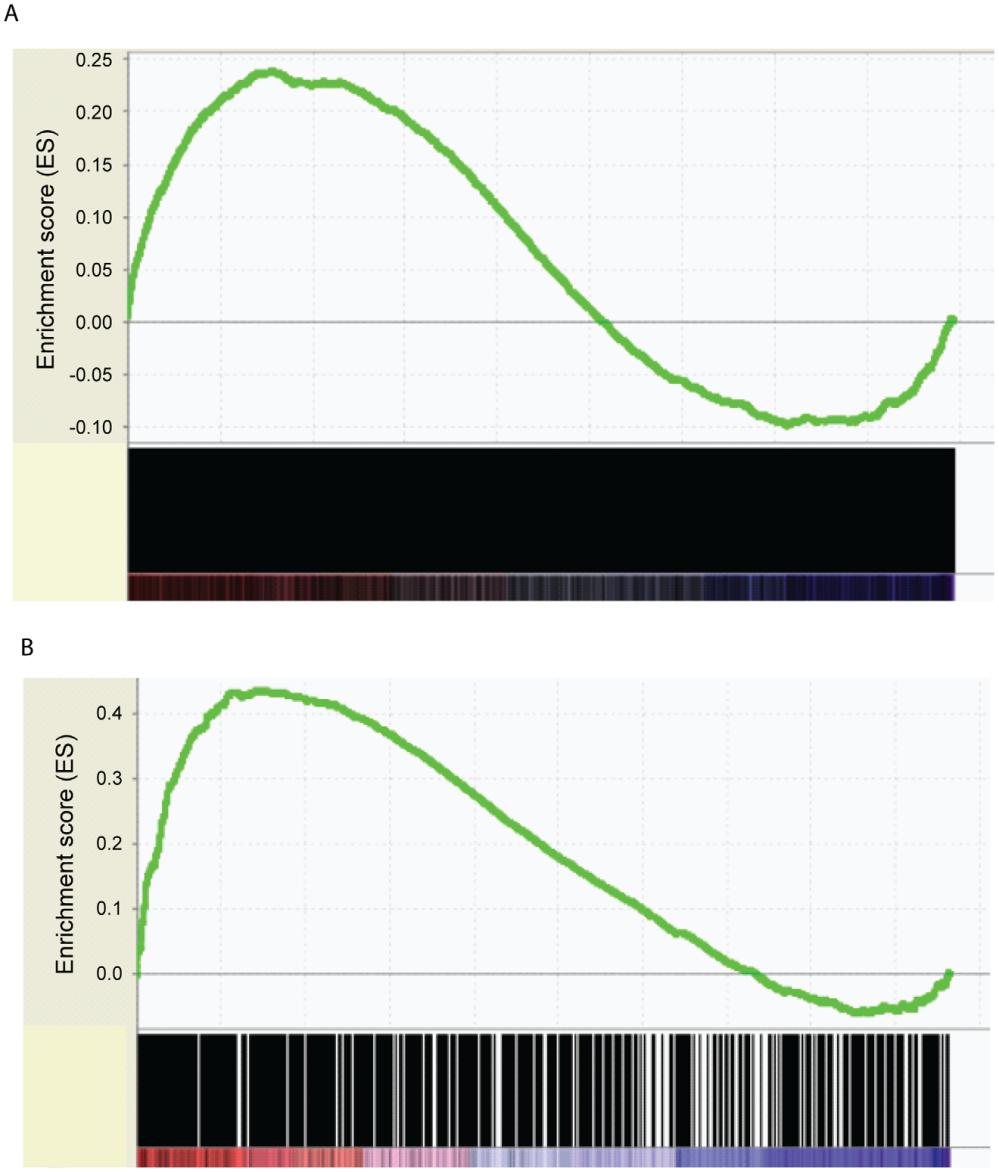
Correlation of transcription factor binding targets with RNA–Seq differential gene expression data by GSEA. (A). A statistically significant enrichment of TCF7 targets was shown among up-regulated genes in CD34+ cells (Nominal p-value = 0, FDR q-value = 0.02). Differential gene expression was ranked by fold change between Lin-CD34+ cells and Lin-CD34− cells (x-axis). The most up-regulated genes in CD34+ cells are shown on the left side (red), while the most up-regulated genes in CD34− cells were shown on the right side (blue). Black bars represent the positions of genes in the ranked list. Enrichment score (ES, Y-axis) reflects the degree to which TCF7 binding targets are overrepresented at the extremes of the ranked gene expression list. When the distribution is at random, the enrichment score is zero. Enrichment of TCF7 targets at the top of the ranked list results in a large positive deviation of the ES from zero. (B) A statistically significant enrichment of RUNX1 targets was shown among up-regulated genes in CD34+ cells (Nominal p-value = 0, FDR q-value = 0). Enrichment of RUNX1 targets at the top of the ranked list results in a large positive deviation of the ES from zero.

To determine how the differential gene expression in CD34+ and CD34− cells is related to TCF7 regulation, Gene Set Enrichment Analysis (GSEA) [37] was performed to correlate transcription factor binding information with the gene expression data. The GSEA software is designed to determine whether members of a gene set, for example TCF7 binding targets, are randomly distributed throughout the gene expression data or primarily enriched among genes most highly up or down-regulated during the switch from CD34+ to CD34− cells. The expression dataset was rank-ordered by fold change such that the most up-regulated genes in CD34+ cells were at the top of the ranked list, while most up-regulated genes in CD34− cells (down-regulated genes in CD34+ cells) were at the bottom of the ranked list. GSEA analysis showed a statistically significant enrichment (*P* near 0) of TCF7 targets among up-regulated genes in CD34+ cells, in comparison to the distribution expected at random (Figure 4A). Overall, these observations strongly indicate that TCF7 primarily binds to genes up-regulated in CD34+ cells. A similar enrichment was observed for RUNX1 binding targets (Figure 4B) so that both of these factors predominantly bind to genes up-regulated in CD34+ cells.

### *Tcf7* shRNA gene inhibition confirms that *Tcf7* is necessary to maintain cells in the undifferentiated state

**Figure 5:**
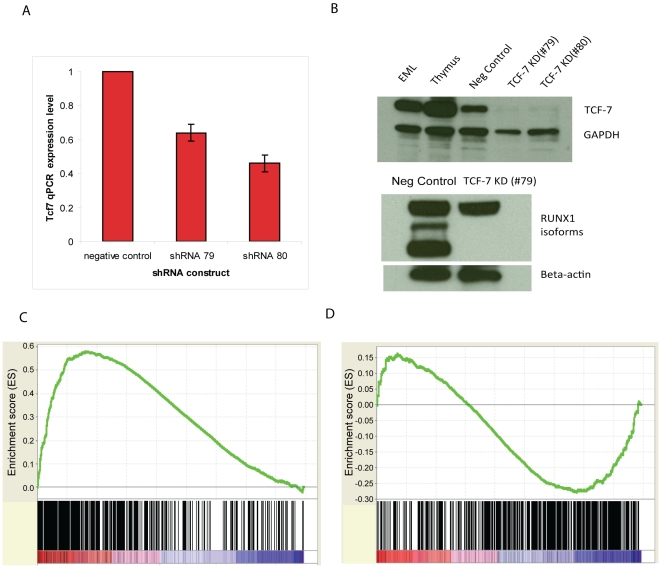
*Tcf7* shRNA gene knockdown experiments provide functional evidence. (A) qRT-PCR validation of the effect of three *Tcf7* shRNA knockdown constructs. *Tcf7* shRNA constructs were shown in the figure using the last two digits of their product numbers: TRCN0000012678, TRCN0000012679, TRCN0000012680. (B) Western blot analysis in TCF7 knockdown cell lines shows TCF7 protein and the shorter isoforms of RUNX1 are absent. Scrambled shRNA serves as a negative control. The polyclonal RUNX1 antibody recognizes the three major isoforms of RUNX1 ranging from ∼25 kDa to ∼50 Kda. The anti actin antibody indicates equal loading. *Tcf7* shRNA knockdown reduces cell proliferation in SCF (scrambled shRNA serves as a negative control). (C) The down-regulated genes in *Tcf7* shRNA knockdown cells are significantly enriched among up-regulated genes in CD34+ cells (Nominal p-value = 0, FDR q-value = 0). (D) The up-regulated genes in *Tcf7* shRNA knockdown cells are significantly enriched among up-regulated genes in CD34− cells (Nominal p-value = 0, FDR q-value = 0.01898734). See Figure 4 legend for GSEA plot details.

To further understand how *Tcf7* affects CD34+ cell self renewal or differentiation, we used shRNA constructs targeting different regions of the *Tcf7* gene. Three *Tcf7* shRNA constructs caused significant reduction in levels (36–54%) of gene expression as confirmed by qRT-PCR experiments and Western blots (Figure 5A, 5B), and we observed obvious effects from the knockdown experiments. Consistent with the hypothesis that *Runx1* and *Tcf7* act coordinately, knockdown of *Tcf7* caused the short isoforms of RUNX1 to disappear at the protein level, without changing the expression of the long isoform (Figure 5B). Illumina bead microarray analysis of cells with reduced expression using one of the constructs revealed that 1510 genes changed >1.5 fold in *Tcf7* knockdown lines compared to a scrambled shRNA negative control line (711 down-regulated and 799 up-regulated genes)(Microarray data is available in the Gene Expression Omnibus (GEO) microarray data repository; record GSE30068). Gene Set Enrichment Analysis (GSEA) was performed to correlate these differentially expressed genes in *Tcf7* knockdown lines with the differential gene expression between CD34+ and CD34− cells. We found that the down-regulated genes in *Tcf7* shRNA knockdown cells are significantly enriched among up-regulated genes in CD34+ cells (Figure 5C; *P* near 0). On the other hand, the up-regulated genes in *Tcf7* shRNA knockdown cells are significantly enriched among up-regulated genes in CD34− cells (Figure 5D; *P* near 0). Therefore, the gene expression profile of *Tcf7* knockdown cells shifted toward a partially differentiated CD34− state. Overall these results suggest that *Tcf7* is necessary for maintaining cells in the undifferentiated CD34+ state but is also necessary for switching to the partially differentiated CD34− state.

### TCF7 and RUNX1 regulate a transcriptional regulatory network that is involved in hematopoietic stem cell establishment and development, cell growth control, and multipotency

**Figure 6:**
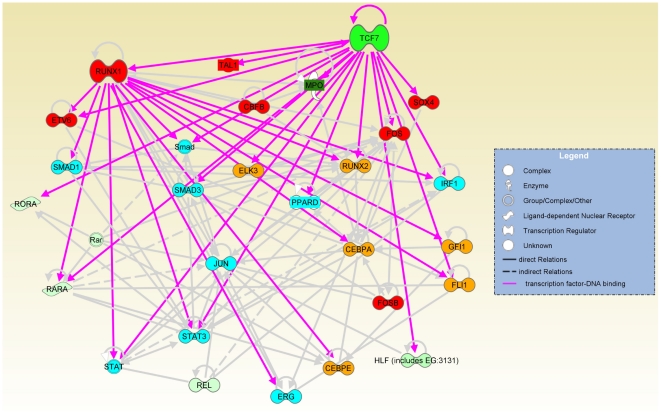
TCF7, together with RUNX1, regulates a transcriptional regulatory network. The network involved in HSC establishment and development (red nodes), cell growth control (blue nodes) and multipotency (orange nodes) was identified among up-regulated genes in CD34+ cells (>2 fold) and displayed by Ingenuity Pathway Analysis software (IPA). Gray lines are IPA annotated relations based on the literature. Pink lines indicate TCF7 or RUNX1 binding to gene targets that were identified by our ChIP-Seq experiments. The shades of green color of the nodes in the network indicate the level of up-regulation in CD34+ cells. *Sox4*, *Mpo*, *Tal1* and *Ppard* were TCF7 binding targets that were added to the network manually because of their obvious interesting function in hematopoiesis and self-renewal. All other nodes were from default IPA analysis. Direct relations were indicated by solid line or arrows. Indirect relations were indicated by dotted line. Please see Ingenuity Pathway Analysis software (IPA https://analysis.ingenuity.com/) Online Help section for detailed definitions.

To understand the intricate relationship of the transcription regulators defining the state of CD34+ cells, we employed Ingenuity Pathway Analysis software (IPA) to identify a network among up-regulated transcription factors in CD34+ cells (>2 fold) (Figure 6). TCF7 and RUNX1 and many transcription factors bound by TCF7 or RUNX1 are found in this network. Overall, three main functional groups can be identified: 1). HSC establishment and development during early hematopoiesis (marked in red in Figure 6). This group includes Sox4, Fos, Tal1 and Etv6. Fos and Sox4 were identified as novel nuclear factors that affect hematopoietic stem cell activity. Overexpression of Fos and Sox4 induced enhanced HSC activity and resulted in an increased repopulating activity compared to the untreated cells [38]. Tal1/Scl is required during the establishment of primitive and definitive haematopoiesis, and plays a role in erythromyeloid lineage commitment [39], [40]. Etv6/Tel is shown to regulate postembryonic HSC survival and is essential for multilineage haematopoiesis in the bone marrow (Hock et al. 2004). CBFB (core-binding factor, beta subunit) is the binding partner of RUNX1. 2). Cell growth control (marked in blue in Figure 6). This group includes Stats, Ppard, Erg and the Tgfβ signaling pathway components Smads etc. 3). Multipotency (marked in yellow in Figure 6). The multipotency of CD34+ cells is reflected in genes involved in lineage specification. For example, Cebpa is a regulator of myelomonocytic lineage commitment. Gfi1 promotes GMP (common myeloid progenitor) differentiation towards the neutrophilic lineage [40]. ETS transcription factor FLI-1 interacts with RUNX1-containing multiprotein complexes through protein-protein interactions and is involved in the transcriptional regulation of megakaryocyte maturation (Huang et al. 2009). Upon differentiation into the CD34−state, there is a remarkable down-regulation of genes for other lineage specifications except for erythroid differentiation. This is consistent with the fact that CD34− cells can no longer proliferate in SCF alone; they depend on an additional cytokine, IL-3, for growth. Therefore, TCF7, together with RUNX1, controls a transcriptional regulatory network determining the choice between EML cell self-renewal of multipotent cells and differentiation. The silencing of *Tcf7* in the CD34− cells would contribute to the commitment to differentiation.

## Discussion

In this study, we identified TCF7 as a regulator in the decision of EML multipotential hematopoietic precursor cell self-renewal and differentiation. We further identified RUNX1 as a partner and effector of TCF7 function. TCF7 and RUNX1 bind to a significantly overlapping set of target genes and likely function coordinately in regulating target genes. In particular, TCF7 and RUNX1 bind to and potentially regulate a network of transcription factors which characterize the gene expression pattern of CD34+ cells. We validated our hypothesis using functional tests.

*Tcf7* is a member of the T-cell factor family of transcription factors that are the downstream effectors of the Wnt signal transduction pathways. Wnt signaling inhibits the degradation of beta-catenin protein by preventing its phosphorylation by GSK3 beta. In the absence of phsophorylation of N-terminal serine and threonine residues, beta-catenin accumulates and is translocated into the nucleus where it associates with members of the TCF family of transcription factors, and furnishes them with a transcriptional activation domain [20]. Wnt signaling can act in a context dependent manner to either activate or repress transcription [41]. The TCF family of transcription factors can also either activate or repress the transcription of genes responsive to Wnt signaling [18].

Wnt signaling has been implicated in self-renewal of hematopoietic stem cells and the growth of hematopoietic precursors [21], [22], [23], [24] although mice with defects in the Wnt signaling pathway continue to develop normal mature cells of the hematopoietic system. Although *Tcf7* knockout mice have only been reported to show a defect in thymocyte development [42], [43], there are multiple TCF family members *in vivo* which can have redundant function and compensate for the loss of *Tcf7* alone in the knock-out mouse model. In the EML system the expression level of other TCF family members are low and none of them displayed as remarkable differential gene expression as observed of *Tcf7*. An analysis of the repopulating activity of the subpopulations of cells in *Tcf7*−/− mouse bone marrow could be useful in the future to understand TCF7's roles in vivo; but such an analysis can be complicated by the stimuli from multiple signaling pathways in the bone marrow and the stem cell niche.

In addition to TCF7's role in thymocyte development, T-lineage specification and differentiation [42], [43], [44], the present study shows TCF7 plays a role in the EML model of hematopoietic precursor cell differentiation and function; and has a role in gene activation as well as repression, A number of components of the Wnt signaling pathway, such as *Lrp5* and *Bcl9*, were expressed at higher levels in CD34+ than CD34− cells. However, the data presented here suggest that regulation by Wnt molecules is not a significant factor in EML cell growth and differentiation. RNA-Seq showed only low levels of the Wnt receptors frizzled 2, 5 and 7, and, at most, traces of Wnt9a and 10a mRNA in the CD34+ cell population, as well as a lack of mRNA for other known Wnt molecules in these cells. Even the trace amounts of *Wnt9a* and *10a* mRNA seen by RNA-Seq in CD34+ cells could not be detected by RT-PCR. *Wnt9a* and *10a* mRNAs were completely absent from the CD34− cells. EML cells are grown in conditioned medium and it is possible that this medium contributed some Wnt. However, EML cells also grow well in standard medium in the presence of only purified SCF so that Wnt in the conditioned media does not seem to be a necessary factor for the growth of the cells. Finally, when a *Tcf/Lef*-GFP reporter was introduced into the EML cells, there was no GFP signal of Wnt induced activity in these cells, although the cells did respond to LiCl or external WNT ligands. We conclude that TCF7 may function in these cells by a pathway operative without autocrine Wnt signaling.

The present study suggests that TCF7 plays a dual role in global gene network by promoting the expression of large number of genes characteristic of self-renewing CD34+ cells (Figure 5C), while repressing genes activated in partially differentiated CD34− state (Figure 5D). The effects of *Tcf7* knock-down is a result of a combination of both direct effects of losing TCF7 binding and secondary effects of removal of the short form of RUNX1 or other TCF7 targets. RUNX1 plays multiple roles in early hematopoietic development and, unlike TCF7 or Wnt, is necessary for emergence of hematopoietic stem cells [45]. In sea-urchin embryos RUNX1 expression is linked to Wnt activity [46]. In EML cells, TCF7 and RUNX1 bind to one another's promoter and thus may regulate each other. The *Runx1* gene encodes both short and long isoforms, and these have antagonistic effects. The short isoforms promote maintenance and proliferation of progenitor cells, but the long isoforms promote differentiation and inhibit progenitor cell repopulation [47]. Remarkably, knockdown of *Tcf7*, caused the short isoforms of RUNX1 to disappear at the protein level, without changing the expression of the long isoforms. Therefore, TCF7, in addition to direct effects on the transcription of individual genes, may prevent differentiation by regulating the relative abundance of RUNX1 isoforms which have opposing effects on differentiation.

There may be additional unknown factors besides TCF7 and RUNX1 that are central for switching between the two cell types. Examination of the TCF7 binding targets whose expression is altered by *Tcf7* inhibition showed that a STAT3 motif was one of the most frequently detectable transcription factor binding motifs (Figure S5). Interestingly STAT3 was one of the transcription factors that are up-regulated in the CD34+ cells, suggesting that increased STAT3 levels might augment TCF7 mediated transcriptional changes in the CD34+ cells. Among other transcription factors whose mRNA levels are higher in CD34+ than in CD34− cells, *Bcl9* and *Jun* are known modulators of the TCF7-beta-catenin transcriptional response. Interestingly, analysis of the binding targets of SCL/TAL1 in a stem/progenitor cell line HPC-7 indicates that they largely overlap with TCF7 binding genes (163 out of 243 SCL target genes are in common with TCF7 targets) [48]. *Scl/Tal1* is one of the TCF7 target genes; however, *Tcf7* is not among the SCL/TAL1 target genes. Therefore, SCL/TAL1 may be a downstream mediator of TCF7 activation. In another context *Bcl3* has been reported to be increased by SCF signaling [49], which may also contribute to the increase in *Bcl3* mRNA levels seen in CD34+ as compared to CD34− cells. Overall, these results suggest that a major part of the transcriptional switch between CD34+ and CD34− cells is mediated by a small network of transcriptional mediators, with TCF7 central to the network. Finally, when *Tcf7* level is knocked down by shRNA, although the transcription levels of many genes changed as mentioned earlier in this paper, CD34+ RNA is not reduced by the knockdown. Therefore, there may be additional transcription regulatory factors required for the switch and this is a topic of our future investigation.

Many models of regulated stem cell differentiation, such as sperm and egg production, skin regeneration, intestinal cell regeneration, and neural differentiation, involve control of the choice of differentiation versus precursor renewal that depends on contact or signaling to the stem cell from different types of cells in anatomically circumscribed niches. The EML system provides a clear example of a mammalian precursor cell that has the intrinsic ability to produce a quantitatively balanced ratio of renewing versus differentiated progenies. We have found that TCF7 is a regulator of the self-renewal and differentiation switch and further analysis of how it is controlled will be critical for understanding how this important process is regulated.

## Materials and Methods

### MACS separation of CD34+ and CD34− cells

Suspension cultures of EML cells were maintained in SCF containing medium as previously described [13]. Total EML cells were washed twice in FACS buffer (0.5% BSA, 1 mM EDTA, 1× PBS) and resuspended in 40 µl FACS buffer per 1×10^7^ cells. 15 µl of Mouse Lineage Depletion Cocktail biotin conjugated antibody (Miltenyi Biotec) was added to the cells for 20 minutes at 4°C. The labeled cells were washed twice in FACS buffer and resuspended in 80 µl FACS buffer. 20 µl of paramagnetic microbeads conjugated to anti-biotin antibody (Miltenyi Biotec) was added to the cells and incubated for 20 minutes at 4°C. The labeled cells were washed twice and resuspended in 2 ml FACS buffer per 1×10^7^ cells. The cells were separated twice in an AutoMACS cell separator (Miltenyi Biotec) using the depletion program (0.5 mls per minute). The lineage negative (Lin-) fraction was resuspended in 100 µl (per 1×10^7^ cells) of FACS buffer and CD34 biotin conjugated antibody was added (1 µg per 1×10^6^ cells). The labeled cells were washed in FACS buffer as above and bound to anti-biotin coated beads. The cells were separated in an AutoMACS cell separator (Miltenyi Biotec) using a double positive separation program. The subsequent Lin-CD34− fraction was resorted with the AutoMACS separator using the depletion program. Lin-CD34+ and Lin-CD34+ cells were collected. The cell purity was checked after each separation using FACS and only cell purity >90% was used for further experimentation.

### FACS separation of CD34+ and CD34− cells

Total EML cells were washed twice in FACS buffer (0.5% BSA, 1 mM EDTA, 1× PBS) and resuspended in 100 µl FACS buffer per 1×10^6^ cells. CD34-FITC (1 µg per 1×10^6^ cells; Ebiosciences) was added to the cells and incubated for 1 hour at 4°C. Sca1-PE (0.06 µg per 1×10^6^ cells; Ebiosciences) and Lineage Cocktail APC (5 µl per 1×10^6^ cells; Miltenyi Biotec) were added to the cells and incubated for an additional 30 minutes. Lin-SCA+CD34+ and Lin-SCA-CD34− cells were collected using FACS Aria (Beckman).

### Illumina RNA–Sequencing and analysis

mRNA samples were prepared from 2×10^6^ CD34+ and CD34− cells. RNA-Seq was performed as described [50]. Two biological replicas and two technical replicas were used for each cell type. The mouse genome sequence, annotation and genomic features (genes, cDNAs, 3′ UTRs, 5′ UTRs, introns, exons, intergenic regions, ESTs) for the mm9 database release were directly downloaded from UCSC Table Browser (http://genome.ucsc.edu) or obtained from Galaxy (http://galaxy.psu.edu/). Raw Illumina reads were obtained after base calling in the Solexa Pipeline version 0.2.2.6. RNA-Seq reads were mapped to the mouse genome using Illumina's ELAND software. Differentially expressed gene features were identified using the ERANGE package [51]. Read coverage along the annotated transcription units was calculated using the ShortRead package [52].

Repetitive mapped reads were combined with uniquely mapped reads to produce a final RPKM (reads per kilobase of mRNA, per million total reads), using the procedure defined for ERANGE, by calculating the probability that a multiread came from a particular known or candidate exon based on the distribution of counts of uniquely mapped reads in each exon. The resulting fractional counts were added to the total count for the gene locus, which was renormalized into a multi RPKM (Gene expression values: Dataset S1).

Transcription factors were identified from the list provided by Luscombe et al [53] of human transcription factors. Homologous mouse genes were obtained for each from the Ensembl database using the biomaRt package. We identified genes with different expression levels in CD34+ versus CD34− cells as those with at least a two fold difference in RPKM and in which both cell types had a minimum of 2 RPKM.

### ChIP–Sequencing

ChIP-Seq was performed as described [54], [55], [56]. 5×10^7^ formaldehyde cross-linked Lin-CD34+ and Lin-CD34− cells were used. TCF7 goat polyclonal antibody TCF1(H18) (Santa Cruz Biotechnology: catalog#SC8589), anti-AML(RUNX1) rabbit polyclonal antibody [36] (CalBiochem: catalog#PC284), and monoclonal RNA PolII antibody (Covance: catalog#8WG16-MMS-126R) were used. IP-western experiments were done to ensure the specificity of the antibodies.

### ChIP–Seq data analysis

ChIP-Sequencing data were analyzed using the PeakSeq program as previously described [57]. The transcription factor binding loci were extracted with statistically significant signals (q-value<0.001). Subsequently, we mapped the binding sites to RefSeq genes in UCSC mm9 database (genome.ucsc.edu). A gene was designated as the target gene if the peak was present within 3000 nt upstream of the transcription start site or inside of the gene (including both exonic and intronic regions). ChIP-Seq data have been deposited to GEO database (GSE31221; reviewer access link: http://www.ncbi.nlm.nih.gov/geo/query/acc.cgi?token=lxglzumiewawcha&acc=GSE31221.) In TCF7 and RUNX1 coregulation analysis, known motifs for each of TCF7 and RUNX1 were obtained from Jaspar and Transfac databases. We random selected 600 sequences bound in TCF7 and RUNX1 ChIP-Seq experiments, and performed de novo binding motif search. We compared these de novo binding motifs with the known motifs (from Jaspar and Transfac databases) of RUNX1 and TCF7.

### Gene Ontology (GO) analysis, Gene Set Enrichment Analysis (GSEA), and Ingenuity Pathway Analysis (IPA)

We used BiNGO [35] to determine the statistically over-represented (p-value<0.0001) Gene Ontology (GO) categories within the target gene sets of the transcription factors, and then visualized the relationships of these GO categories with the Cytoscape software [58].

We also performed Gene Set Enrichment Analysis (GSEA) [37] to correlate transcription factor binding information with the gene expression data. The expression dataset was rank-ordered by fold change (difference of classes ranking metric) such that the most up-regulated genes in Lin-CD34+ cells were on the top of the ranked list, while the most up-regulated genes in Lin-CD34− cells (down-regulated genes in Lin-CD34+ cells) were at the bottom of the ranked list. GSEA analysis was used to determine whether members of a binding target list, are randomly distributed throughout the gene expression data or primarily enriched toward the top or bottom of the gene expression list using the default weighted enrichment statistic.

Ingenuity Pathway Analysis (IPA) was performed to display the transcription factor regulatory networks (http://www.ingenuity.com/).

### shRNA knockdown of *Tcf7* in EML cells

Fresh EML cells were recovered one week prior to shRNA experiments in SCF containing growth medium (GM) as previously described [13]. 1.5×10^4^ EML Lin-CD34+cells were double FACS sorted and infected with shRNA constructs containing *Tcf7* (Sigma Aldrich) at an MOI = 2 in a round bottom 96 well plate. Five shRNA constructs targeting different regions of the *Tcf7* gene were used (Sigma, shRNA product numbers: TRCN0000012678, TRCN0000012679, TRCN0000012680, TRCN0000012681, TRCN0000012682). SHC002VMISSION Non-Target shRNA Control Transduction Particles (Sigma) were used as shRNA negative control. To increase transduction efficiency the ExpressMag systems (Sigma Aldrich) was used according to manufacturer's instructions. 24 hours post transduction, cells were selected in EML GM containing puromycin (1.2 µg/ml). When indicated, selected cells were grown in expansion medium (IMDB, 20% heat inactivated horse serum, 100 ng/ml SCF [PeproTech]). Cells were analyzed for knockdown efficiency using qPCR (*Tcf7* forward primer sequence ATCCTTGATGCTGGGATTCTG; *Tcf7* reverse primer sequence CTTCTCTTGCCTTGGGTTCTG. CD34 forward primer sequence aggctgatgctggtgctagt; reverse primer sequence ccccagctttctcaagtcag. Two internal controls: HPRT forward primer tatgccgaggatttggaaaa; HPRT reverse primer acagagggccacaatgtgat, and/or beta Actin forward primer gatctggcaccacaccttct; reverse primer accagaggcatacagggaca). In addition, Western blot analysis was performed on puromycin-selected *Tcf7* knockdown cell lines to examine TCF7 and RUNX1 protein expression. The polyclonal TCF7 antibody (Sigma Aldrich, catalog#AV34782) and RUNX1 antibody (Abcam, catalog# ab23980) were used. The anti actin (Abcam, catalog#ab8229) antibody was used to indicate equal loading.

For Illumina array analysis, Lin- CD34+cells (1×10^5^) cells were transduced at an MOI = 1 with a *Tcf7* targeting shRNA construct TRCN0000012679 or a shRNA negative control. After a 24-hour incubation with the shRNA-containing virus, the cells were grown in EML GM for 24 hours, then cells were selected for 24 hours in puromycin (1.2 µg/ml). Cells were harvested (a total of four days after initial sort) and total RNA extracted. Total RNA from *Tcf7* shRNA knockdown cells and control cells (transfected with scrambled shRNA) was purified using the RNeasy Plus kit from Qiagen. Hybridization to Illumina Mouse WG-6 v2.0 Expression BeadChips was conducted at the Stanford Functional Genomics Facility using standard Illumina protocols. The microarray data was processed using the R version 2.11 Bioconductor Lumi package. The gene expression values were offset so that all values were made positive, subjected to the VST variance stabilization transformation, and were then quantile normalized. Z scores are plotted where Z = (x−μ)/σ, x is the log2 transformed gene expression measurement and μ and σ are the mean and standard deviations of expression of the gene. The microarray data is in compliance with MIAME guidelines. The data have been deposited in GEO database (GSE30068).

### Monitoring endogenous Wnt signaling in EML cells

To test for the presence of *wnt10a* mRNA, four sets of PCR primers were designed cross introns, which could distinguish between genomic DNA and spliced cDNA.

Wnt10a forward primer 1:GCGCTCCTGTTCTTCCTACT, Wnt10a reverse primer 1: GATCTGGATGCCCTGGATAG; Wnt10a forward primer 2: GGCGCTCCTGTTCTTCCTAC, Wnt10a reverse primer 2: ATGCCCTGGATAGCAGAGG; Wnt10a forward primer 3: CATGAGTGCCAGCATCAGTT, Wnt10a reverse primer 3: ACCGCAAGCCTTCAGTTTAC; Wnt10a forward primer 4: CATGAGTGCCAGCATCAGTT, Wnt10a reverse primer 4: AGCCTTCAGTTTACCCAGAGC.

Total EML cells (5×10^4^) were infected with lentivirus containing either a CMV-GFP construct or a *Tcf/Lef*-GFP construct (SABiosciences) at a MOI of 1, 5, 10, and 20. After 24 hours, cells were selected in EML GM with puromycin (1.2 µg/ml). Selected cells were expanded in EML GM for 4–6 days. Cells were incubated for 24 hours in either LiCL (50 mM), WNT3a (400 uM, PeproTech) or WNT5a (400 um, PeproTech). Cells were analyzed by FACSCalibur (BD Biosciences).

## Supporting Information

Figure S1 Analysis of the DNA sequence motifs that were enriched among up-regulated genes in CD34+ cell. DIRE analysis shows binding motifs of the TCF family of transcription factors (TF) are among the DNA sequence motifs that were enriched in up-regulated genes (>1.5 fold) in CD34+ cell. The pie chart indicates the distribution of the locations of the potential regulatory element. 1.5 kb upstream of the transcription start site is considered as a promoter region. TF occurrence: percentage of candidate regulatory elements containing a conserved binding site for a particular TF. TF importance: product of TF occurrence and TF weigh (DiRE optimization procedure calculates a weight for each transcription factor (TF) as a measure of its association with the input gene set) (see details at: http://dire.dcode.org/).
(TIF)

Figure S2 Minimum endogenous Wnt signaling in EML cells detectable with a *Tcf/Lef* GFP reporter system. The *Tcf/Lef* reporter is under the control of a minimum CMV promoter fused in tandem to *Tcf/Lef* transcriptional response elements. A CMV-GFP construct was used as a positive control (upper right panel). The *Tcf/Lef*-GFP construct were used to infect total EML cells at a MOI of 5 and 10 (the middle panels). In a parallel experiment, we incubated EML cells infected with *Tcf/Lef*-GFP construct with LiCl (50 mM) for 24 hours (lower panels). The percentage of cells that show GFP signal is sown in the pink box.
(TIF)

Figure S3 Hierarchical relationships among enriched functional categories of TCF7 binding targets *via* BiNGO. Gene Ontology Analysis shows genes associated with regulation of transcription were highly enriched in TCF7 targets. A P-value cutoff of 1.00E-4 was used to identify significantly enriched nodes. P-values are indicated by a color scale as shown. Node size corresponds to the number of genes within each category. Some category labels are not shown for clarity.
(TIF)

Figure S4 De novo binding motifs overlap well with the known motifs of RUNX1 or TCF7. (A). de novo binding motif derived from TCF7 ChIP-Seq dataset (lower) showed statistically significant overlap (11 nt out of12 nt) with TCF7 known motif (upper) in the same orientation. (B). de novo binding motif derived from RUNX1 ChIP-Seq datasets (lower) showed statistically significant overlap (10 out of 10 nt) with RUNX1 known motif (upper) in the complementary orientation.
(TIF)

Figure S5 STAT3 motif enrichment. Examination of the genes that are affected by *Tcf7* inhibition showed that a STAT3 motif was one of the most frequently detectable transcription factor binding motifs among the genes that are also TCF7 binding targets. (A). The top 10 enriched motifs identified among the up-regulated TCF7 targets genes (>1.5 fold) when *Tcf7* is knocked down; (B). The top 10 enriched motifs among the down-regulated TCF7 targets genes (>1.5 fold) when *Tcf7* is knocked down. STAT3 is marked by a red circle in the list. See the legend of Figure S1 for detailed description of DIRE analysis output.
(TIF)

Table S1 Enriched functional categories among differentially expressed transcription factors in either CD34+ or CD34− cells. IPA analysis: https://analysis.ingenuity.com/).
(TIF)

Table S2 Gene expression values of Wnt pathway components in CD34+ and CD34− cells using RNA-Seq. Standard Deviation was calculated among two biological replicas and two technical replicas for each cell type.
(TIF)

Dataset S1 RNA–Seq gene expression values from each replica of CD34+ and CD34− cells.
(XLS)

Dataset S2 TCF7 binding regions and other information (scored by PeakSeq program, q-value<0.001).
(XLS)

Dataset S3 RUNX1 binding regions and other information (scored by PeakSeq program, q-value<0.001).
(XLS)

Dataset S4 Target gene lists of TCF7 and RUNX1.
(XLS)
