# Supplementary figures and images for: Tcf7 Is an Important Regulator of the Switch of Self-Renewal and Differentiation in a Multipotential Hematopoietic Cell Line
Source: PLoS Genet. 2012 Mar 8;8(3):e1002565. doi: 10.1371/journal.pgen.1002565 (PMC3297581; doi:10.1371/journal.pgen.1002565)

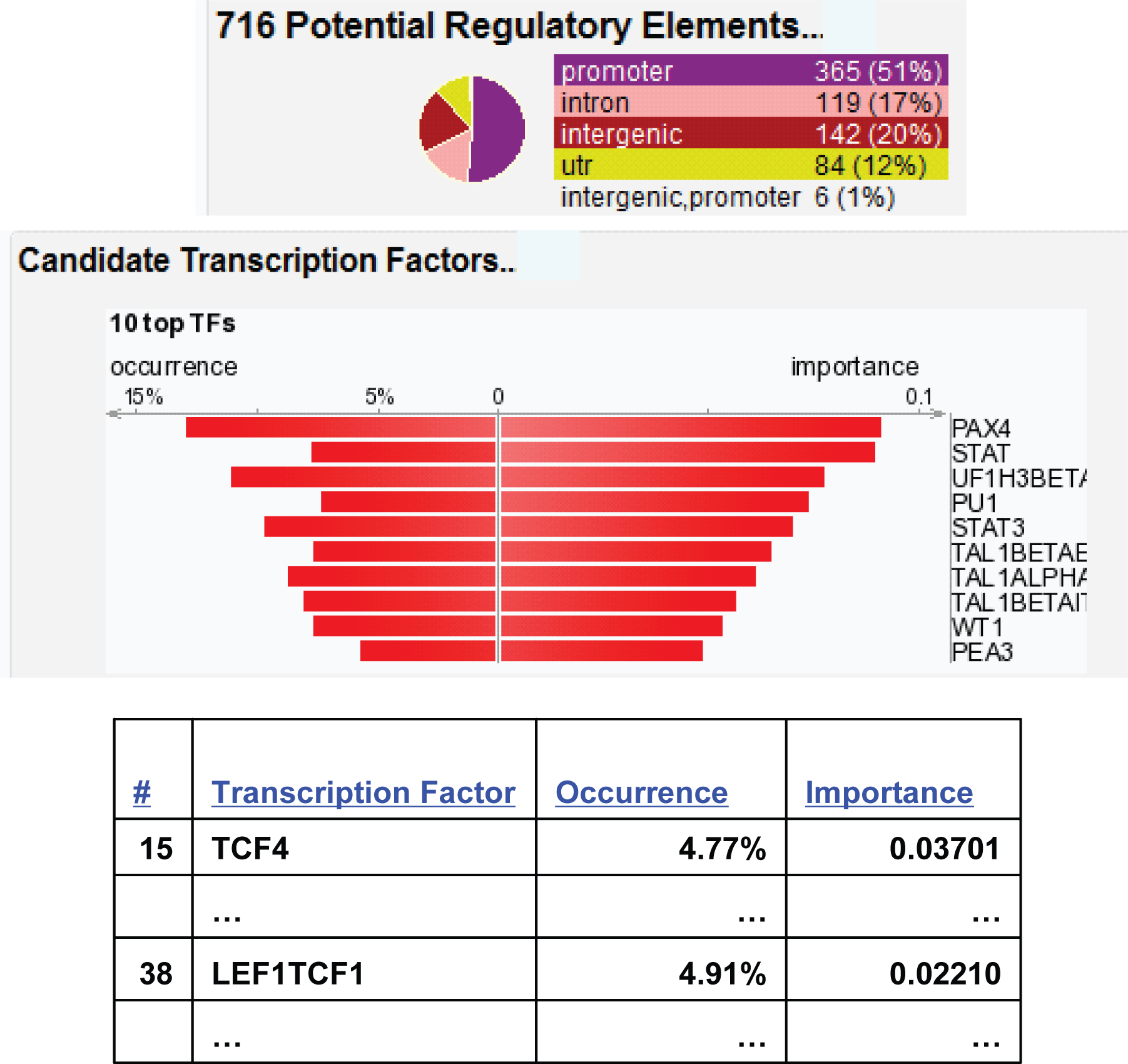

Supplement: Figure S1 — Analysis of the DNA sequence motifs that were enriched among up-regulated genes in CD34+ cell. DIRE analysis shows binding motifs of the TCF family of transcription factors (TF) are among the DNA sequence motifs that were enriched in up-regulated genes (>1.5 fold) in CD34+ cell. The pie chart indicates the distribution of the locations of the potential regulatory element. 1.5 kb upstream of the transcription start site is considered as a promoter region. TF occurrence: percentage of candidate regulatory elements containing a conserved binding site for a particular TF. TF importance: product of TF occurrence and TF weigh (DiRE optimization procedure calculates a weight for each transcription factor (TF) as a measure of its association with the input gene set) (see details at: http://dire.dcode.org/). (TIF) [file pgen.1002565.s001.tif]

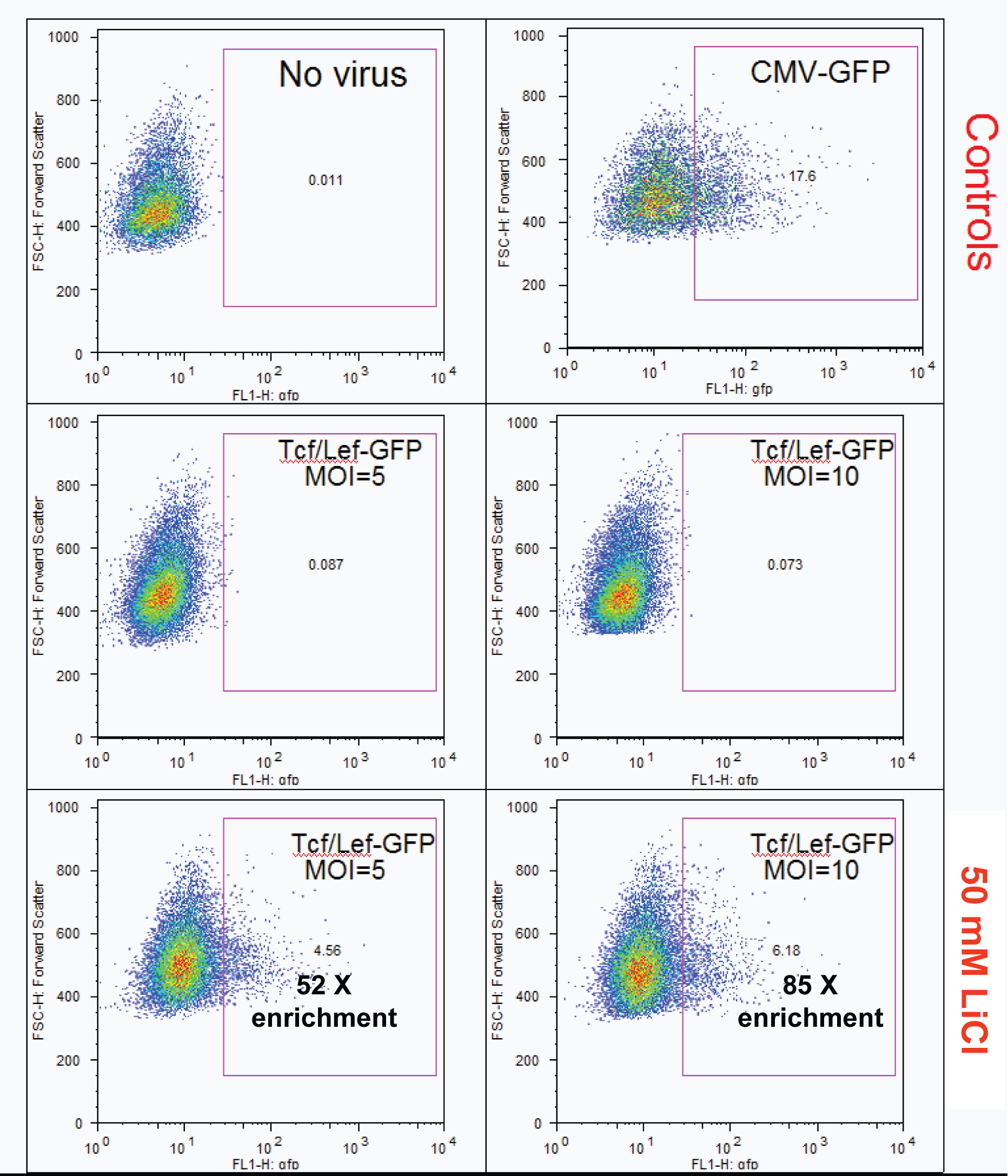

Supplement: Figure S2 — Minimum endogenous Wnt signaling in EML cells detectable with a Tcf/Lef GFP reporter system. The Tcf/Lef reporter is under the control of a minimum CMV promoter fused in tandem to Tcf/Lef transcriptional response elements. A CMV-GFP construct was used as a positive control (upper right panel). The Tcf/Lef-GFP construct were used to infect total EML cells at a MOI of 5 and 10 (the middle panels). In a parallel experiment, we incubated EML cells infected with Tcf/Lef-GFP construct with LiCl (50 mM) for 24 hours (lower panels). The percentage of cells that show GFP signal is sown in the pink box. (TIF) [file pgen.1002565.s002.tif]

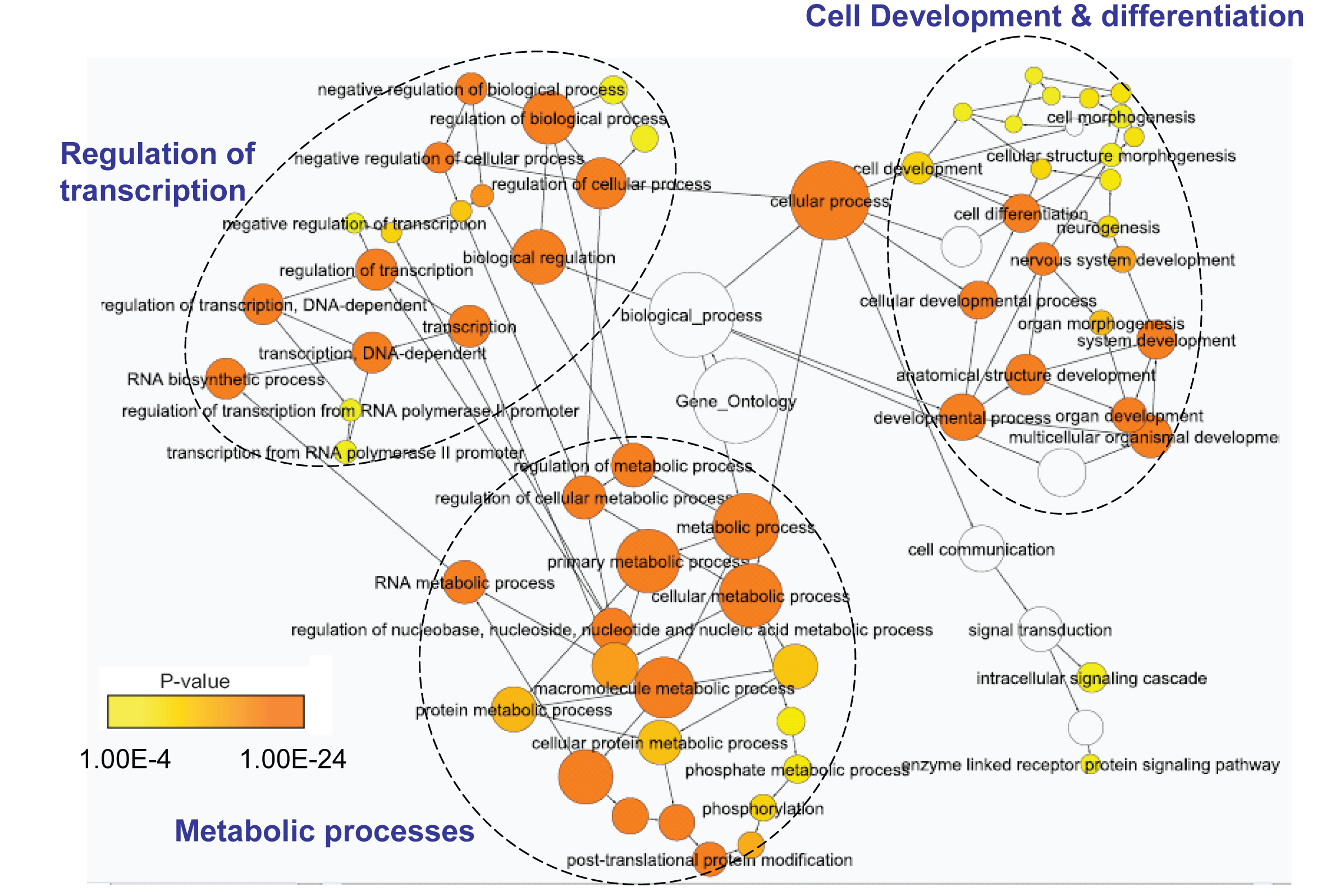

Supplement: Figure S3 — Hierarchical relationships among enriched functional categories of TCF7 binding targets via BiNGO. Gene Ontology Analysis shows genes associated with regulation of transcription were highly enriched in TCF7 targets. A P-value cutoff of 1.00E-4 was used to identify significantly enriched nodes. P-values are indicated by a color scale as shown. Node size corresponds to the number of genes within each category. Some category labels are not shown for clarity. (TIF) [file pgen.1002565.s003.tif]

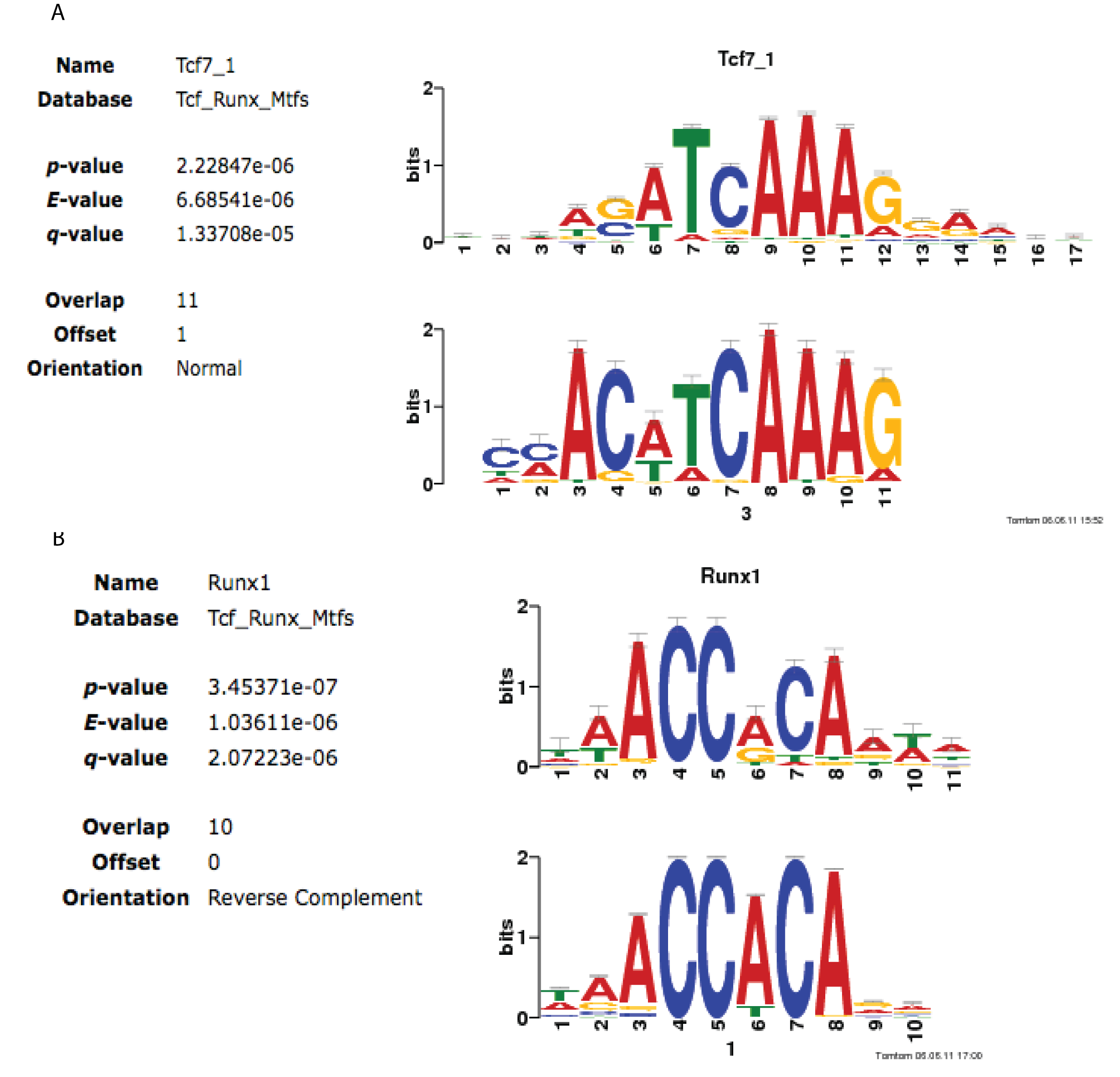

Supplement: Figure S4 — De novo binding motifs overlap well with the known motifs of RUNX1 or TCF7. (A). de novo binding motif derived from TCF7 ChIP-Seq dataset (lower) showed statistically significant overlap (11 nt out of12 nt) with TCF7 known motif (upper) in the same orientation. (B). de novo binding motif derived from RUNX1 ChIP-Seq datasets (lower) showed statistically significant overlap (10 out of 10 nt) with RUNX1 known motif (upper) in the complementary orientation. (TIF) [file pgen.1002565.s004.tif]

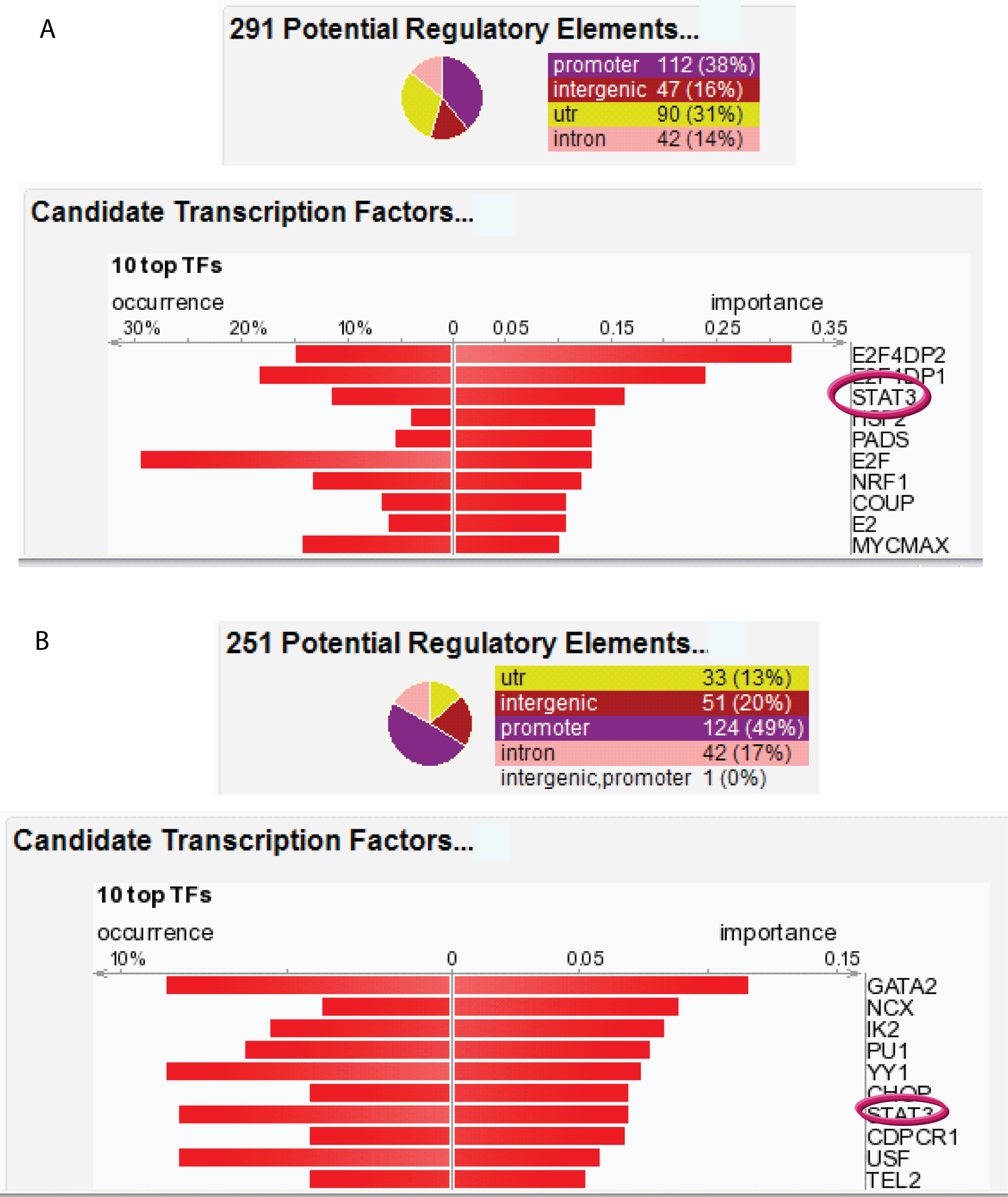

Supplement: Figure S5 — STAT3 motif enrichment. Examination of the genes that are affected by Tcf7 inhibition showed that a STAT3 motif was one of the most frequently detectable transcription factor binding motifs among the genes that are also TCF7 binding targets. (A). The top 10 enriched motifs identified among the up-regulated TCF7 targets genes (>1.5 fold) when Tcf7 is knocked down; (B). The top 10 enriched motifs among the down-regulated TCF7 targets genes (>1.5 fold) when Tcf7 is knocked down. STAT3 is marked by a red circle in the list. See the legend of Figure S1 for detailed description of DIRE analysis output. (TIF) [file pgen.1002565.s005.tif]

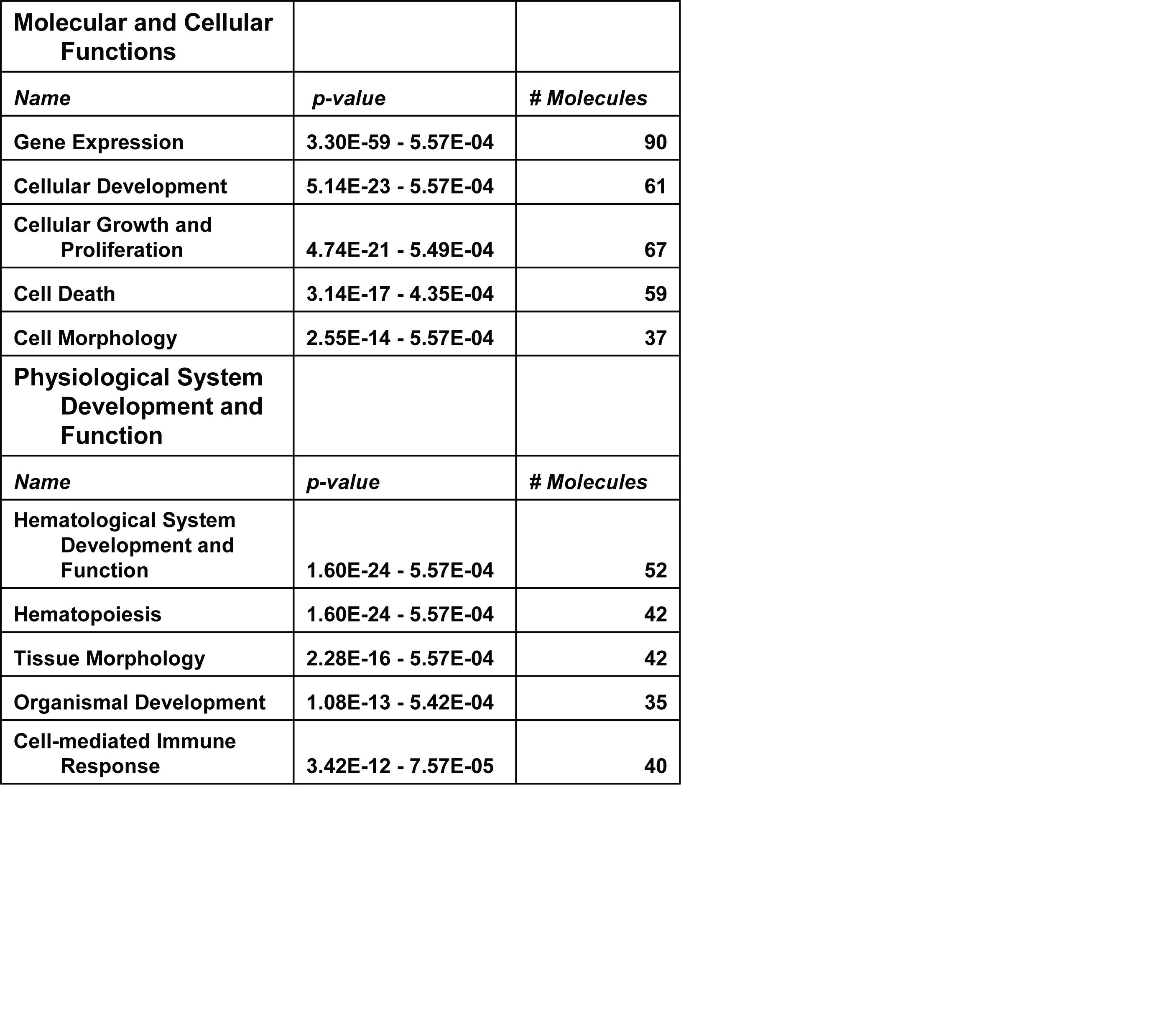

Supplement: Table S1 — Enriched functional categories among differentially expressed transcription factors in either CD34+ or CD34− cells. IPA analysis: https://analysis.ingenuity.com/). (TIF) [file pgen.1002565.s006.tif]

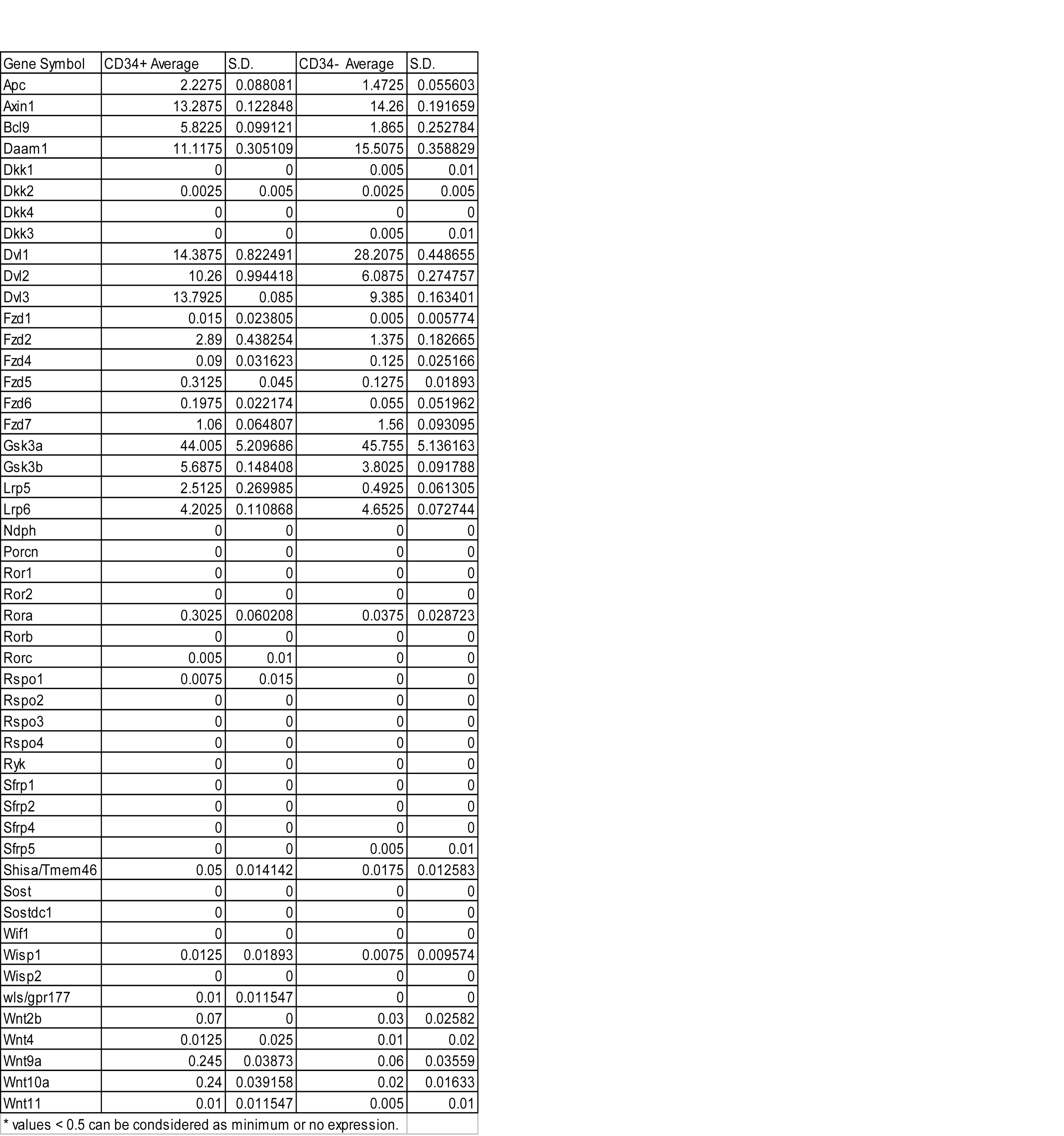

Supplement: Table S2 — Gene expression values of Wnt pathway components in CD34+ and CD34− cells using RNA-Seq. Standard Deviation was calculated among two biological replicas and two technical replicas for each cell type. (TIF) [file pgen.1002565.s007.tif]
